# Single-cell transcriptomics reveals the cellular dynamics of hexafluoropropylene oxide dimer acid in exerting mouse male reproductive toxicity

**DOI:** 10.1186/s40104-025-01177-x

**Published:** 2025-03-11

**Authors:** Xupeng Zang, Yongzhong Wang, Lei Jiang, Yuhao Qiu, Yue Ding, Shengchen Gu, Gengyuan Cai, Ting Gu, Linjun Hong

**Affiliations:** 1 State Key Laboratory of Swine and Poultry Breeding Industry, National Engineering Research Center for Breeding Swine Industry, Guangdong Provincial Key Laboratory of Agro-Animal Genomics and Molecular Breeding, College of Animal Science, South China Agricultural University, Guangzhou, 510642 China; 2 Yunfu Subcenter of Guangdong Laboratory for Lingnan Modern Agriculture, Yunfu, 527300 China; 3 National Regional Gene Bank of Livestock and Poultry (Gene Bank of Guangdong Livestock and Poultry), Guangzhou, 510642 China

**Keywords:** Cellular dynamics, GenX, Reproductive toxicity, Single-cell RNA sequencing, Testis

## Abstract

**Background:**

Hexafluoropropylene oxide dimer acid (GenX), a substitute for per- and polyfluoroalkyl substances, has been widely detected in various environmental matrices and foods recently, attracting great attention. However, a systematic characterization of its reproductive toxicity is still missing. This study aims to explore the male reproductive toxicity caused by GenX exposure and the potential cellular and molecular regulatory mechanisms behind it.

**Results:**

Normally developing mice were exposed to GenX, and testicular tissue was subsequently analyzed and validated using single-cell RNA sequencing. Our results revealed that GenX induced severe testicular damage, disrupted the balance between undifferentiated and differentiated spermatogonial stem cells, and led to strong variation in the cellular dynamics of spermatogenesis. Furthermore, GenX exposure caused global upregulation of testicular somatic cellular inflammatory responses, increased abnormal macrophage differentiation, and attenuated fibroblast adhesion, disorganizing the somatic-germline interactions.

**Conclusions:**

In conclusion, this study revealed complex cellular dynamics and transcriptome changes in mouse testis after GenX exposure, providing a valuable resource for understanding its reproductive toxicity.

**Supplementary Information:**

The online version contains supplementary material available at 10.1186/s40104-025-01177-x.

## Background

Per- and polyfluoroalkyl substances (PFAS) have been widely used in industry and food production since the last century [1, 2]. The increasing production and use of these synthetic chemicals have raised great concern about their environmental and health impacts. Numerous studies have shown that perfluorooctanoic acid (PFOA), one of the most common PFAS, is widely persistent and potentially toxic in the environment. Considering its harmful effects on the environment and humans, the emission and production of PFOA have been gradually restricted worldwide [3, 4]. As a result, alternatives to PFOA are becoming increasingly popular as they are considered to pose lower risks to the environment [5]. Among them, hexafluoropropylene oxide dimer acid (HFPO-DA), commonly known as GenX, is a novel alternative to PFOA [6]. However, over the past decade, GenX has also been widely detected in various environmental matrices around the world, including drinking water and food, causing very high concern among people [7–9].

Recently, there has been growing evidence that GenX poses potentially substantial risks to human and animal health. For example, GenX exposure reduced thyroid cell viability, induced DNA damage, and altered gene expression levels in thyroid cells [10]. While exposure of GenX to fertilized eggs via air cell injection induced developmental cardiotoxicity, including thinning of the right ventricular wall and increased heart rate, and developmental hepatotoxicity in the form of steatosis [11]. Furthermore, GenX exposure can also affect mammalian inflammation, leading to gut barrier dysfunction and gut microbiota disturbance [12].

As the most important organ in the male reproductive system, the testis is highly sensitive to environmental pollutants, and the integrity of its physiological structure is essential for successful spermatogenesis [13]. Although histological studies have shown that GenX exposure can disrupt the testicular blood-testis barrier and cause male reproductive toxicity [14], the cellular and molecular mechanisms by which GenX exposure leads to impaired testicular function remain unclear. This knowledge gap requires a comprehensive investigation of the cellular dynamics influenced by GenX exposure, particularly in the testicular environment.

Therefore, in this study, we employed mice as a model to reveal the complex cellular responses and transcriptomic alterations in the testis exposed to GenX using single-cell RNA sequencing (scRNA-seq), which is powerful in studying testicular development and pathology [15, 16]. We evaluated the damage induced by GenX to the mouse testis, constructed a detailed single-cell transcriptome profile of the testis after GenX exposure, and investigated the corresponding cellular dynamics changes. These results provide a comprehensive understanding of the cellular and molecular dynamics caused by GenX, paving the way for future studies and potential therapeutic interventions to mitigate its reproductive toxicity.

## Materials and methods

### Animals

The animal experiments and procedures involved in this study were approved by the Ethics Committee of the Laboratory Animal Center of South China Agricultural University (permit number: SYXK-2024-0136).

Specific pathogen-free (SPF) 6-week-old male BALB/c mice were randomly divided into 3 groups, with 6 mice in each group. The mice were fed a conventional formula diet and housed in a controlled room at 22 °C with a 12:12 h light–dark cycle. After one week of acclimation, GenX (P888213, Macklin, China) was administered daily by oral gavage at concentrations of 0, 12.5, or 62.5 mg/kg body weight for 28 d. Mice were euthanized and testicular tissue and serum samples were collected on 29 d.

The selection of the oral dose was based on several previous studies on GenX’s biotoxicity [14, 17–20], which is higher than levels found in food and human exposure in the environment. Based on the data from earlier studies on GenX toxicity in CD-1 mice, a no-observed-adverse-effect level (NOAEL) of 5 mg/kg/d for reproductive and maternal systemic toxicity was established. In a study on pregnant rats, Lv et al*.* [17] found that the GenX concentration in the 10 mg/kg/d dose group was the lowest observed effective concentration. Conley et al*.* [20] showed that when rat dams were exposed to doses of GenX below 30 mg/kg/d from day 8 of pregnancy to day 2 after delivery, and there was no effect on fetal birth weight. However, when the GenX exposure concentration was 62.5 mg/kg/d, the mortality rate of the pups increased significantly. Based on the above points, and considering that the equivalent dose in mice is approximately twice that in rats [21], we selected oral doses of 12.5 mg/kg/d and 62.5 mg/kg/d for this study.

### ELISA assay

Testosterone levels in mouse serum were quantified using the ELISA kit (KGE010, R&D Systems, USA) according to the manufacturer’s protocols.

### Analytical chemical determination of GenX concentrations

GenX concentrations in mouse serum were analyzed using a similar method as those reported by Conley et al*.* [20] and Lv et al*.* [17]. Briefly, 50 μL of serum sample was mixed with 300 μL of methanol by vortexing for 10 min and then centrifuged at 15,000 r/min for 10 min at 4 °C. The resulting supernatant was carefully aspirated and diluted tenfold before transfer to the chromatography vials. All samples were prepared in this manner. The GenX standard was designed at 10 concentrations of 0.35, 0.7, 1.4, 3.5, 7, 14, 35, 70, 140 and 350 ng/mL. A standard curve was drawn based on the 10 standard concentrations and used for quantification. The sample extracts were separated using an ExionLC ultrahigh-performance liquid chromatograph coupled to an LTQ-MS (Sciex, Framingham, MA, USA) equipped with an ACQUITY UPLC BEH C18 column (2.1 mm × 50 mm; 1.7 μm), and the column temperature was set at 40 °C.

### Hematoxylin and eosin (H&E) staining

Paraffin-embedded mouse testis samples were sliced into 5 μm thick sections. The sections were stained with hematoxylin and eosin for histological assessment. Nikon 80i microscope was used to observe and photograph the images (Nikon, Japan).

### Periodic acid-Schiff (PAS) staining

Testicular tissue sections were deparaffinized and oxidized in 0.5% periodic acid solution at room temperature for 10 min. Then, sections were rinsed with free-flowing distilled water and incubated with Schiff reagent in the dark for 10 min. The sections were rinsed with free-flowing distilled water again and counterstained with hematoxylin solution for 90 s. Finally, the sections were rinsed, air-dried, and mounted with neutral gum.

### Masson’s trichrome staining

Testicular tissue sections were deparaffinized and incubated overnight with potassium dichromate. Then, sections were incubated sequentially in Weigert’s iron hematoxylin working solution for 5 min, in Ponceau S acid fuchsin staining solution for 5 min, in phosphomolybdic acid solution for 5 min, and in aniline blue for 5 min. After washing in distilled water, the sections were differentiated in 1% glacial acetic acid for 1 min. Finally, the sections were dehydrated and mounted with neutral gum.

### Immunofluorescence staining

Paraffin-embedded mouse testis samples were sliced into 4 μm thick sections. The sections were dewaxed and hydrated, and antigen retrieval was performed with sodium citrate antigen retrieval buffer (pH 6.0). Endogenous peroxides were quenched with 3% H_2_O_2_. Then, sections were blocked with 3% bovine serum albumin (BSA) at room temperature for 30 min, incubated with the primary antibody (Acta2, 67735-1-Ig, Proteintech; Crisp2, 19066-1-AP, Proteintech; Cd68, ab213363, Abcam; Col3a1, GB111629, Servicebio) at 4 °C overnight, and incubated with the corresponding secondary antibody at room temperature for 50 min. Finally, the cell nuclei were counterstained with DAPI.

### Testicular tissue dissociation and single-cell suspension preparation

The mouse testis with the outer capsule removed was minced into small pieces of about 0.5 mm^3^ using surgical scissors. The cells were then dissociated using 0.2 mg/mL Liberase TL solution (Sigma) at 37 °C for 15 min. The dissociated cells were filtered through a 40-μm cell strainer (Corning) and centrifuged at 300 × *g* for 5 min at 4 °C. After removing the supernatant, the pelleted cells were suspended in erythrocyte lysis buffer (MACS) at 4 °C for 10 min to exclude mature erythrocytes. Finally, the cells were washed twice with ice-cold PBS and resuspended in PBS supplemented with 0.04% BSA.

### Single-cell RNA-seq library generation and sequencing

The concentration of single-cell suspensions was adjusted to 700–1,200 cells/μL. Then, single-cell suspensions were loaded on a Chromium Single Cell Controller (10X Genomics) according to the manufacturer’s instructions (Chromium Next GEM Single Cell 3’ Reagent Kit V3.1). All subsequent steps were performed following the standard manufacturer’s protocols. Purified libraries were analyzed by the Illumina NovaSeq 6000 platform using a paired-end 150-bp sequencing strategy.

### Data processing of single-cell RNA-seq data

The 10X Genomics scRNA-seq raw data were processed to quantify gene counts using the Cell Ranger pipeline (v.7.1.0). First, binary base call (BCL) files generated by the Illumina NovaSeq 6000 platform were converted to standard FASTQ files using the mkfastq function in Cell Ranger. The mouse reference genome and gene annotation files were obtained directly from the 10X Genomics website. Subsequently, cell number and gene count in the library were determined using the count function with the parameter “–include-introns = False”. Downstream analyses were performed in the R package Seurat (v.5.0.0) [22].

### Quality control of single-cell RNA-seq data

Considering the highly active characteristics of testicular cells, rigorous quality control standards were established. Specifically, cells expressing at least 1,000 genes and less than 8% of mitochondrial genes were used for subsequent analysis. Only genes expressed by more than three cells were retained for follow-up analysis.

### Dimension reduction and unsupervised clustering

Highly variable genes (HVGs) were calculated using the FindVariableFeatures function with the parameter “nfeature = 5,000”. Then, the datasets of different samples were integrated by the FastMNNIntegration method using the IntegrateLayers function with default parameters in Seurat5.0. 1–40 mnn reduction was determined to perform the RunUMAP function, and the FindClusters function with an appropriate resolution was used to identify testicular cell clusters through clustering based on shared nearest neighbor (SNN) modularity optimization. Testicular cell types were determined based on well-known classical testicular cell-specific markers.

### Differentially expressed genes (DEGs) identification

The FindAllMarkers function based on the Wilcoxon rank sum test was used to identify DEGs between different cell types with the parameters “logfc.threshold = 0.5, min.pct = 0.5, only.pos = TRUE”, as well as based on DESeq2 was used to identify DEGs of each cell type after GenX exposure with the parameters “logfc.threshold = 0.25, min.pct = 0.25, only.pos = TRUE”.

### GO enrichment and KEGG pathway analysis

Gene Ontology (GO) enrichment and Kyoto Encyclopedia of Genes and Genomes (KEGG) pathway analysis of the identified genes were implemented by the R package clusterProfiler (v.4.6.2) [23] and visualized by ggplot2 (v.3.4.1). GO terms and KEGG pathways with a corrected FDR value < 0.05 were defined as significantly enriched.

### Pseudotime trajectory inference

Most cell state transitions, both in development and disease, are characterized by cascades of gene expression changes. In scRNA-seq, a technique called “pseudotemporal ordering” has been developed to order cells along reconstructed differentiation “trajectories” or other types of inferred biological transitions [24]. In this study, the single-cell pseudotime trajectories were predicted using Slingshot (v.2.6.0) [25], monocle3 (v.1.3.1), and monocle2 (v.2.22.0) [26] algorithms with default parameters to discover changes in germ cells and somatic cells in mouse testis, respectively. Slingshot and monocle3 were used to demonstrate the pseudotemporal dynamics of different cell types over development, whereas monocle2 was used to highlight differences in cell differentiation. For monocle2, the eigengenes calculated by the differentialGeneTest function (q value < 0.01) were selected as ordering genes to sort cells into pseudotime order. A discriminative dimensionality reduction with trees (DDRtree) method was used to reduce dimensionality using the reduceDimension function with the parameter “max_components = 2”.

### Cell–cell communication analysis

The interactions between various cell types in mouse testis were inferred using the built-in mouse database and standard workflow in CellChat (v.1.6.1) [27]. Cell–cell communication analysis was performed for testicular cells of GenX-exposed and control mice, respectively. Briefly, CellChat objects of GenX-exposed and control mice were instantiated using the createCellChat function, respectively. Then, the corresponding intracellular overexpressed ligands or receptors and their interactions were identified using identifyOverExpressedGenes and identifyOverExpressedInteractions functions. Next, communication probabilities were inferred using computeCommunProb and computeCommunProbPathway functions, and the integrated cell communication network was calculated using the aggregateNet function. Finally, the results of GenX-exposed and control cell communication were merged using the mergeCellChat function and visualized.

## Results

### GenX induced testicular damage in mice

**Fig. 1:**
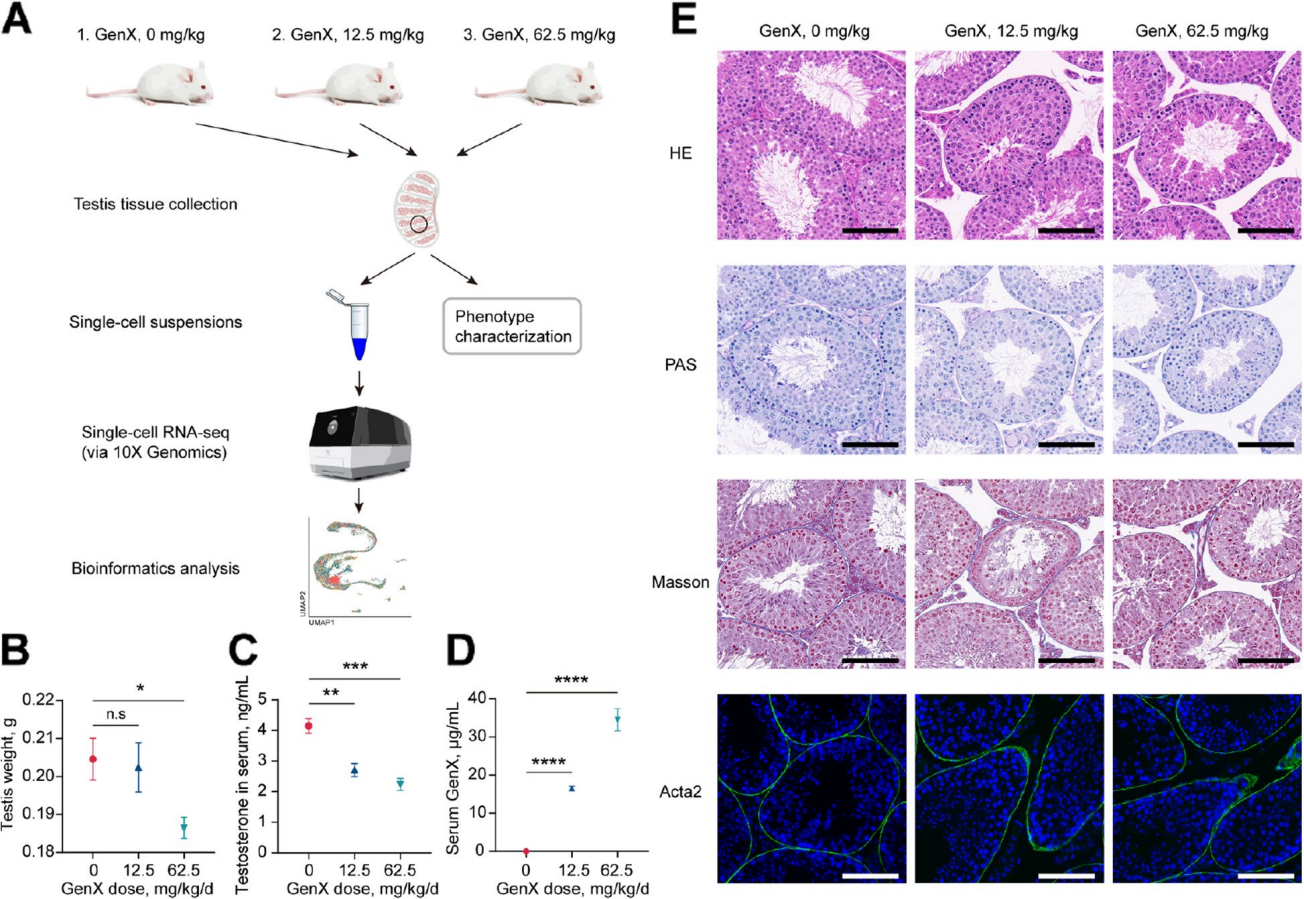
Phenotypic characterization of testicular damage in mouse testis after GenX exposure. **A** Schematic diagram of the experimental workflow of this study. **B** Testicular weight of mice after exposure to different GenX concentrations (*n* = 6 per group). Data was represented as mean ± standard error of the mean (SEM). *P < 0.05 vs. control. **C** Testosterone levels in mouse serum after GenX exposure (*n* = 6 per group). Data was represented as mean ± SEM. **P < 0.01 and ***P < 0.001 vs. control. **D** GenX concentration in mouse serum after GenX exposure (*n* = 6 per group). Data was represented as mean ± SEM. ****P < 0.0001 vs. control. **E** Hematoxylin and eosin (H&E), Periodic acid-Schiff (PAS), Masson’s trichrome, and Acta2 protein immunofluorescence staining of mouse testis tissue sections. Scale bar = 100 μm

To investigate the effects of GenX exposure on male reproductive toxicity, this study treated normal developing male mice using different concentrations of GenX and found that the testicular weight and serum testosterone levels of male mice decreased with increasing GenX doses (Fig. 1A–C). In contrast, the serum GenX concentration increased with increasing exposure doses (Fig. 1D). The average serum GenX concentration of mice in the 12.5 mg/kg/d group was 16.53 μg/mL, and in the 62.5 mg/kg/d group was 34.48 μg/mL. The testicular weight of mice exposed to 62.5 mg/kg/d GenX was significantly lower than that of the control group (Fig. 1B). Although the testicular weight of mice exposed to 12.5 mg/kg/d GenX did not change significantly compared with the control group, their serum testosterone levels, which is essential for maintaining the integrity of the blood-testis barrier and spermatogenesis [28], were reduced considerably (Fig. 1C). Histologically, after GenX exposure, disordered spermatogenic cell layers and vacuolization, increased extracellular matrix deposition in the interstitial tissue, and an impaired blood-testis barrier were observed (Fig. 1E), consistent with previous reports [14]. Taken together, exposure to GenX can cause testicular-related damage.

### Construction of a single-cell transcriptome profile of mouse testis after GenX exposure

**Fig. 2:**
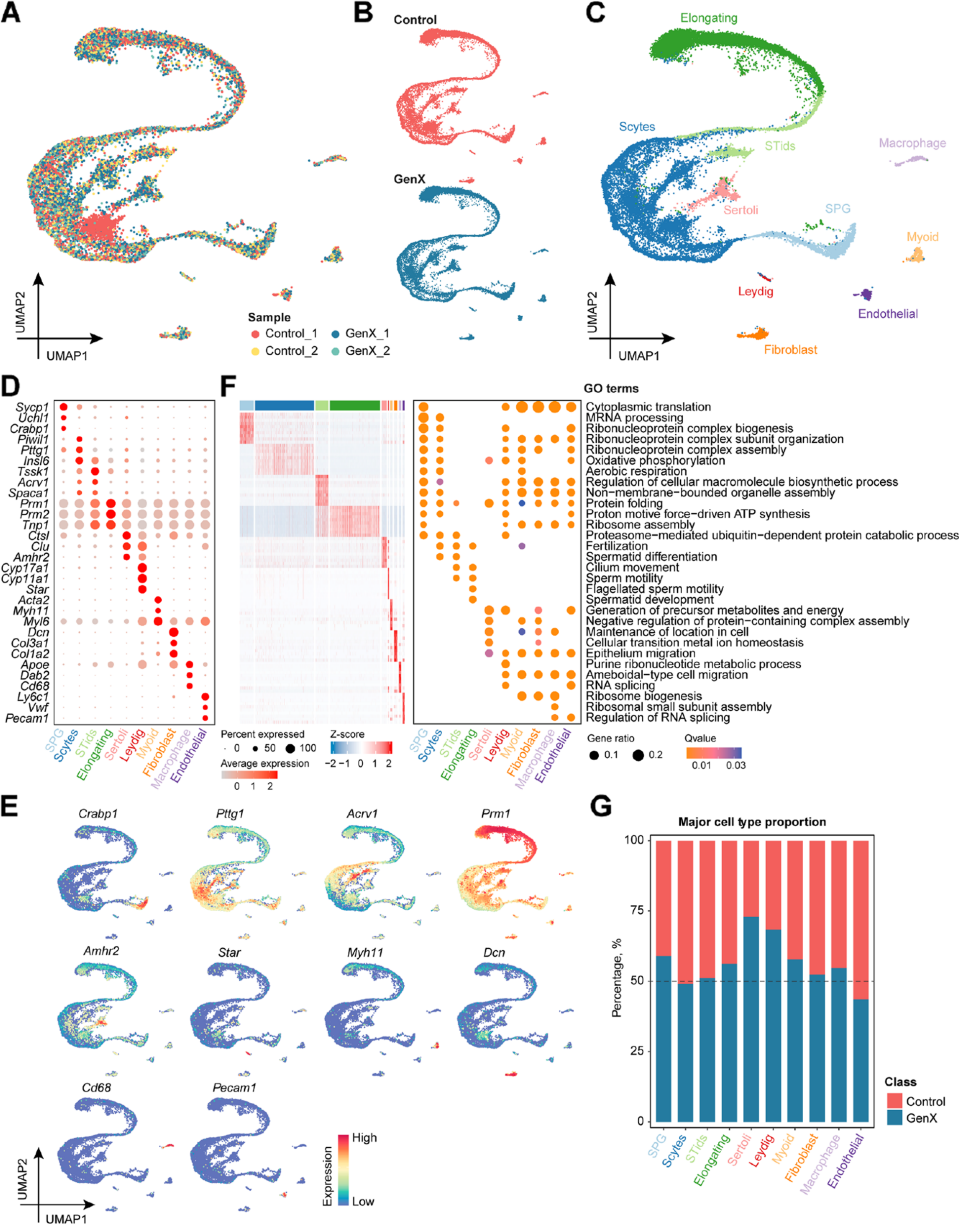
Single-cell transcriptome profile of mouse testis after GenX exposure compared with the control group. **A** UMAP visualization showing testicular cells from different mouse testis samples after GenX exposure. Each dot represents a testicular cell, which is colored by different samples. **B** UMAP visualization showing testicular cells of GenX-exposed and control groups, respectively. **C** UMAP visualization showing the ten testicular cell types in mouse testis. Cells from different mouse testes are colored by cell type. **D** Dot plot of canonical marker genes for mouse testicular cell types. **E** UMAP visualization showing selected marker gene expression in different testicular cell types. **F** Heatmap showing the top 10 differentially expressed genes (DEGs) for each cell type, with each row represents a gene and each column represents a cell. The dot plot on the right showing the GO biological process terms enriched by the corresponding cell type DEGs. **G** Stacked bar plot showing the changes in cell proportions in mouse testis after GenX exposure. 100% is the total number of each cell type identified in GenX-exposed and control testis samples. SPG, spermatogonia; Scytes, spermatocytes; Stids, round spermatids; Elongating, elongating spermatids

To understand the potential mechanism behind GenX-induced testicular damage, we performed scRNA-seq of testicular samples from mice exposed to 62.5 mg/kg/d GenX and control using the 10X Genomics Chromium platform (Fig. 1A). Testis samples from 4 mice, 2 mice from each 62.5 mg/kg/d GenX and control group, were randomly selected for scRNA-seq. After strict quality control, a total of 25,928 cells (12,024 cells from control and 13,904 from GenX) were retained for subsequent analysis (Fig. 2A and B). Unsupervised clustering and identification of cell clusters were performed by uniform manifold approximation and projection (UMAP), and typical marker genes were used to identify four major types of germ cells, including spermatogonia (SPG), spermatocytes (Scytes), round spermatids (STids) and elongating spermatids (Elongating), and 6 somatic cell types, including Sertoli cells, Leydig cells, myoid cells, fibroblasts, macrophages and endothelial cells (Fig. 2C–E, Additional file 1). The DEGs of each cell type and the corresponding GO enriched terms related to their biological functions further confirmed the assigned cell types (Fig. 2F). Notably, preliminary comparisons indicated that certain changes occurred in the proportion of cells in the mouse testis after GenX exposure (Fig. 2G), and detailed analysis is needed to understand their cellular dynamics.

### Strong variation in cellular dynamics of spermatogenesis in mice after GenX exposure

**Fig. 3:**
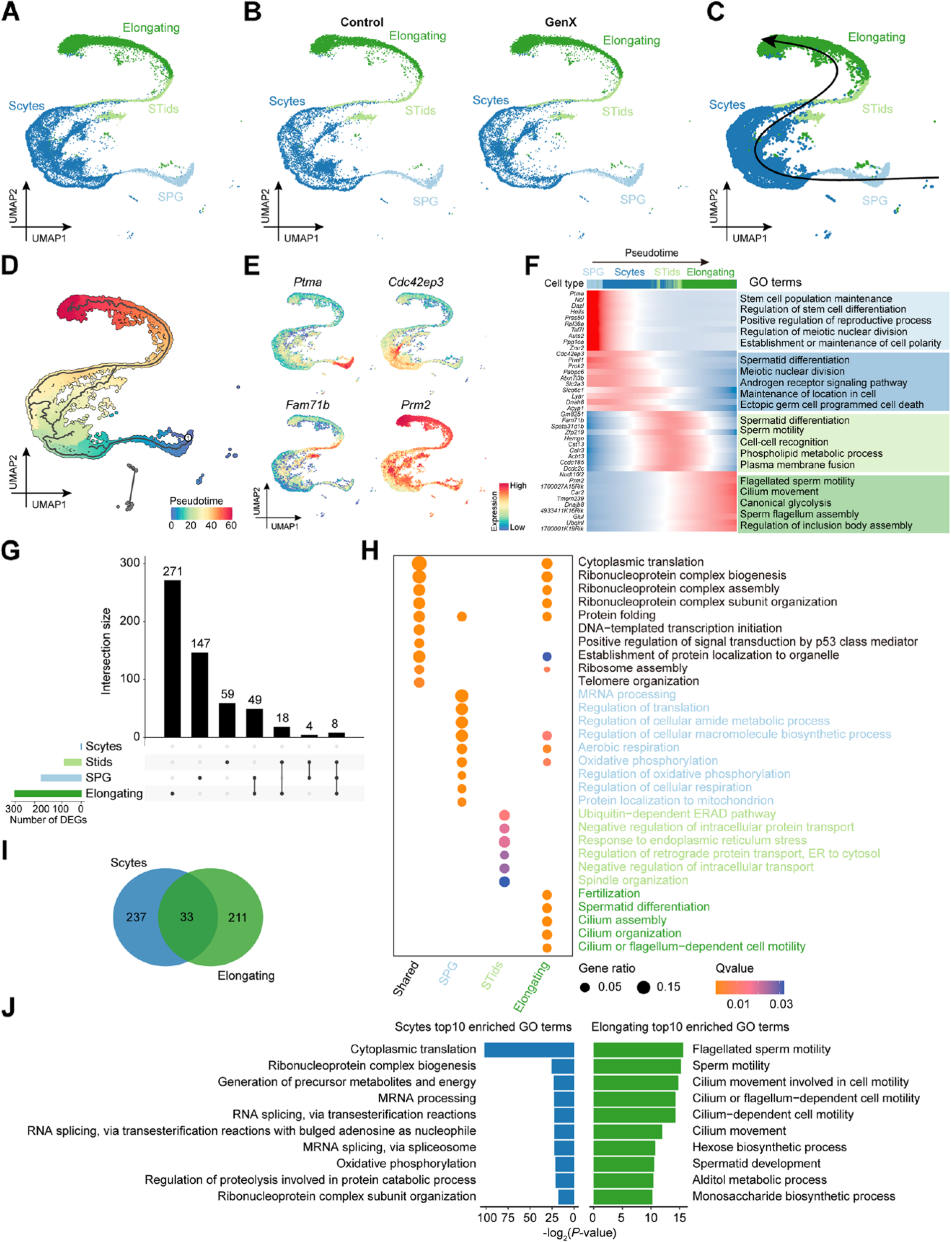
Cellular and molecular characteristics after GenX exposure of germ cells in mouse testis. **A**–**F** The analysis was performed on all germ cells of GenX-exposed and control mice. **A** UMAP visualization showing distinct germ cell types in mouse testis. Cells from different mouse testes are colored by germ cell type. **B** UMAP visualization showing different types of germ cells in GenX-exposed and control groups, respectively. **C** Spermatogenesis trajectories in mouse testis predicted using Slingshot. The black curve representing the predicted pseudotime trajectory and the arrow representing the direction. **D** Predicted spermatogenesis trajectories colored with pseudotime score using Monocle3. **E** UMAP visualization showing selected marker gene expression in different germ cell types. **F** Heatmaps showing the gene expression profiles of the top 10 DEGs in four germ cell types along the spermatogenesis trajectory, with blue indicating low levels and red indicating high levels of expression. Each row represents a gene and each column represents a cell. Representative GO biological process terms enriched by the corresponding cell types DEGs were shown on the right. **G**–**J** Differential gene expression analysis of each germ cell type after GenX exposure. **G** UpSet plot showing common and unique up-regulated DEGs among germ cells in mouse testis after GenX exposure. **H** Top GO biological process terms enriched for up-regulated DEGs in germ cells after GenX exposure. **I** Venn plot showing the distribution of down-regulated DEGs in Scytes and Elongating cells after GenX exposure. **J** Top GO biological process terms enriched for down-regulated DEGs in Scytes and Elongating cells after GenX exposure

To dissect the cellular dynamics of germ cells after GenX exposure, we focused on the identified mouse germ cells from Fig. 2C (Fig. 3A). Further examination of the distribution of germ cell types after GenX exposure did not reveal significantly altered spermatogenesis (Fig. 3B). Pseudotime analysis of all germ cells in GenX-exposed and control mice showed that these germ cells developed step by step in the order of SPG, Scytes, STids, and Elongating (Fig. 3C and D). The DEGs between different types of germ cells showed dynamic changes, and the corresponding GO biological process terms were enriched specifically in cell types along the pseudotime (Fig. 3E and F, Additional file 2). For example, SPG DEGs showed a sharp decrease along the pseudotime and were enriched in biological processes such as “stem cell population maintenance” and “regulation of stem cell differentiation”. The DEGs of Scytes were enriched for biological processes associated with “spermatid differentiation” and “maintenance of location in cell”, while the DEGs of Elongating were enriched in “flagellated sperm motility” and “cilium movement”. These results highlight the essential roles of these biological processes in male germ cell development.

To further explore the potential molecular mechanisms of testicular damage after GenX exposure, we identified and compared DEGs in different types of germ cells along the spermatogenesis trajectory (Fig. 3G–J). Multiple germ cell types showed specific and shared up-regulated DEGs (Fig. 3G). GO enrichment analysis of these DEGs revealed that shared up-regulated DEGs were related to “cytoplasmic translation” and “ribosome assembly”, SPG up-regulated DEGs were associated with “aerobic respiration” and “oxidative phosphorylation”, STids up-regulated DEGs were enriched in “ubiquitin-dependent ERAD pathway” and “response to endoplasmic reticulum stress”, and Elongating up-regulated DEGs were enriched for “spermatid differentiation” and “cilium assembly” (Fig. 3H). Interestingly, down-regulated DEGs were only identified in Scytes and Elongating (Fig. 3I). The down-regulated DEGs of Scytes were also related to “cytoplasmic translation” and “oxidative phosphorylation”, while the Elongating down-regulated DEGs were enriched in “sperm motility” and “cilium movement” (Fig. 3J). Notably, Elongating exhibited the largest number of DEGs after GenX exposure (Fig. 3G and I), indicating that GenX may have the greatest impact on mature sperm.

### GenX disrupted the balance of undifferentiated spermatogonial stem cells (SSCs) and differentiated SSCs in mouse testis

**Fig. 4:**
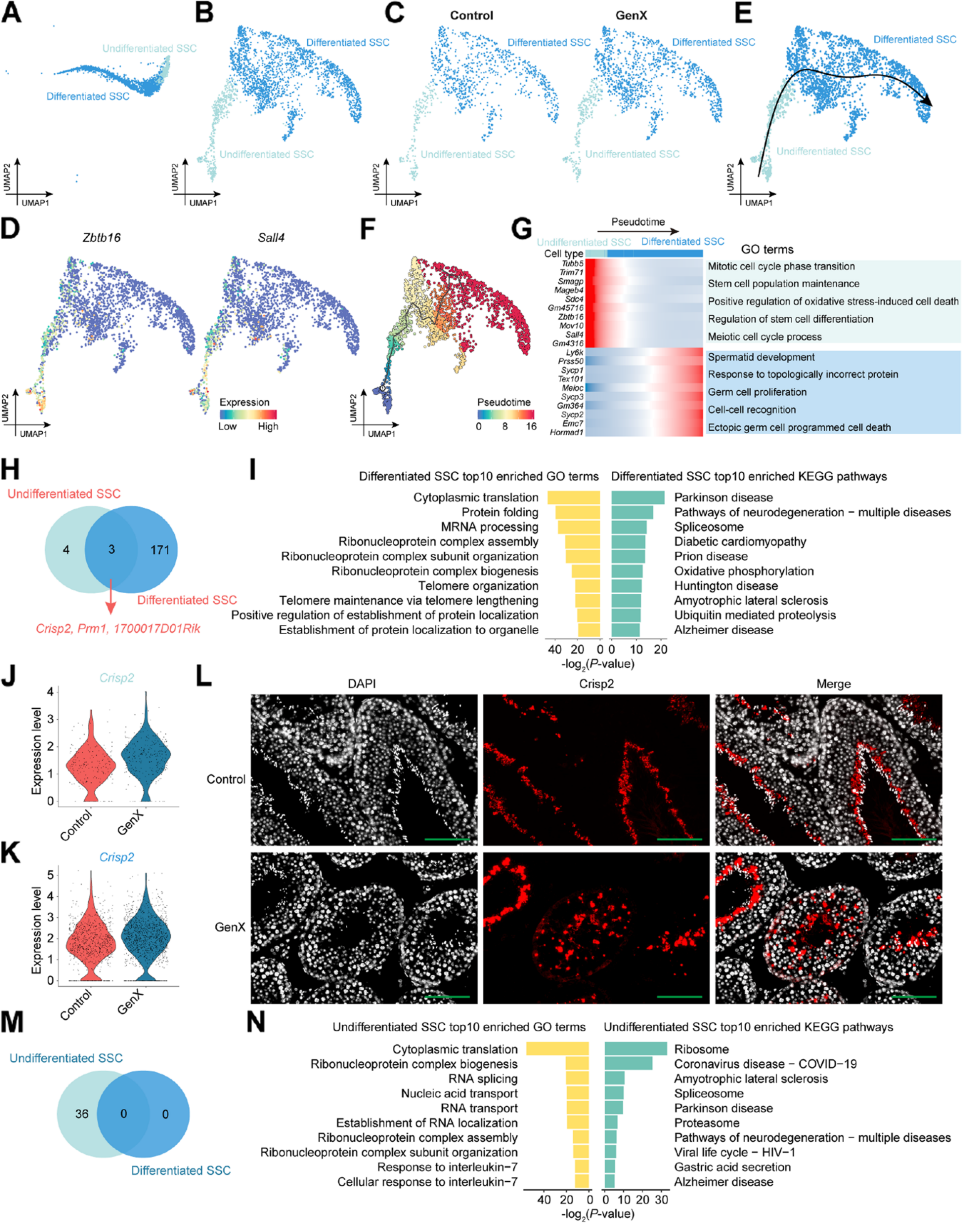
Cellular and functional changes in SPG after GenX exposure. **A**–**G** The analysis was performed on all SPGs of GenX-exposed and control mice. **A** UMAP visualization of SPGs in mouse testis. Cells from different mouse testes are colored by SPG subclusters. **B** UMAP visualization showing the re-clustered SPG subclusters in mouse testis. **C** UMAP visualization showing different SPG subclusters in the GenX-exposed and control groups, respectively. **D** UMAP visualization showing the expression of selected marker genes in different SPG subclusters. **E** SPG differentiation trajectories in mouse testis predicted using Slingshot. The black curve representing the predicted pseudotime trajectory and the arrow representing the direction. **F** Predicted SPG differentiation trajectories colored with pseudotime score using Monocle3. **G** Heatmap showing the gene expression profiles of the top 10 DEGs in two SPG subclusters along the SPG differentiation trajectory, with blue indicating low levels and red indicating high levels of expression. Representative GO biological process terms enriched by the corresponding cell types DEGs were shown on the right. **H**–**N** Differential gene expression analysis of undifferentiated SSCs and differentiated SSCs after GenX exposure. **H** Venn plot showing the distribution of up-regulated DEGs in undifferentiated SSCs and differentiated SSCs after GenX exposure. **I** Top GO biological process terms and KEGG pathways enriched for up-regulated DEGs in differentiated SSCs after GenX exposure. **J** Violin plot showing the expression level of Crisp2 gene in undifferentiated SSCs after GenX exposure compared with the control group. **K** Violin plot showing the expression level of *Crisp2* gene in differentiated SSCs after GenX exposure compared with the control group. **L** Immunofluorescence staining of Crisp2 protein in mouse testis tissue sections. Scale bar = 100 μm. **M** Venn plot showing the distribution of down-regulated DEGs in undifferentiated SSCs and differentiated SSCs after GenX exposure. **N** Top GO biological process terms and KEGG pathways enriched for down-regulated DEGs in undifferentiated SSCs after GenX exposure

The balance between SPG self-renewal and differentiation is crucial for maintaining spermatogenesis homeostasis [29]. Based on previous studies [30], this study further subdivided SPG into undifferentiated SSCs and differentiated SSCs (Fig. 4A–C), among which undifferentiated SSCs expressed SSC typical marker genes *Zbtb16* and *Sall4* (Fig. 4D). Pseudotime analysis of all SPGs in GenX-exposed and control mice showed that SPG developed step by step in the order from undifferentiated SSCs to differentiated SSCs (Fig. 4E and F). The DEGs between undifferentiated SSCs and differentiated SSCs showed dynamic changes, and the corresponding GO biological process terms were specifically enriched along the pseudotime (Fig. 4G). For example, in undifferentiated SSCs, DEGs decreased sharply along the pseudotime and were enriched in biological processes such as “stem cell population maintenance” and “regulation of stem cell differentiation”, while in differentiated SSCs, DEGs were enriched in “spermatid development” and “germ cell proliferation”.

We further confirmed the changes in molecular events during the transition from undifferentiated SSCs to differentiated SSCs after exposure to GenX. Compared with undifferentiated SSCs, differentiated SSCs exhibited a large number of up-regulated DEGs (Fig. 4H). These DEGs were enriched in biological processes related to “cytoplasmic translation”, “protein folding”, and “telomere organization” (Fig. 4I). Three of the DEGs overlapped in undifferentiated SSCs and differentiated SSCs, including *Crisp2*, *Prm1*, and *1700017D01Rik* (Fig. 4H). Crisp2 is an important sperm protein in spermatogenesis, flagellar motility regulation and acrosome reaction, and is indispensable for the function of mature sperm [31, 32]. The premature increase in *Crisp2* expression levels indicated SPG dysfunction after GenX exposure (Fig. 4J–L). Interestingly, no down-regulated DEGs were found in differentiated SSCs, so we only performed functional enrichment analysis on the DEGs down-regulated in undifferentiated SSCs (Fig. 4M). Among them, the “response to interleukin-7” caught our attention (Fig. 4N). Inflammatory signals in the microenvironment can promote the maintenance of stem cell stemness while inhibiting differentiation [33]. The down-regulation of related gene expression levels after GenX exposure suggested potential damage to undifferentiated SSCs. Taken together, these results suggest that the changes associated with GenX exposure disrupt the balance between undifferentiated SSCs and differentiated SSCs to some extent.

### Global changes in testicular somatic cell dynamics in mice after GenX exposure

**Fig. 5:**
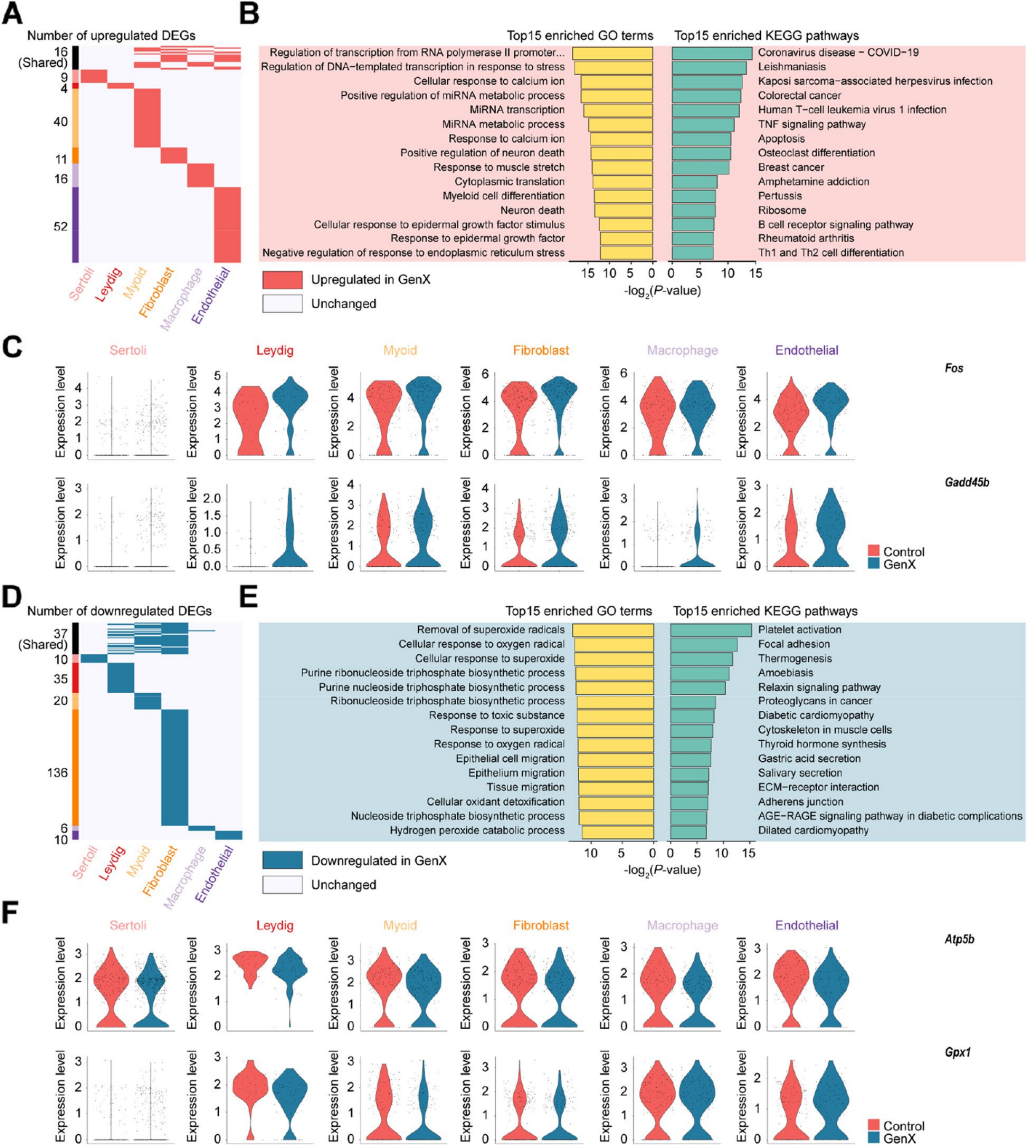
Cellular and molecular characteristics after GenX exposure of somatic cells in mouse testis. **A** Heatmap showing common and unique up-regulated DEGs in each mouse testicular somatic cell type after GenX exposure compared with the control group. **B** Top GO biological process terms and KEGG pathways enriched for shared up-regulated DEGs among somatic cells after GenX exposure. **C** Violin plots showing up-regulated DEGs in mouse testicular somatic cells associated with apoptosis after GenX exposure. **D** Heatmap showing common and unique down-regulated DEGs in each mouse testicular somatic cell type after GenX exposure. **E** Top GO biological process terms and KEGG pathways enriched for shared down-regulated DEGs among somatic cells after GenX exposure. **F** Violin plots showing down-regulated DEGs in mouse testicular somatic cells associated with cell migration after GenX exposure

To understand the cellular and molecular events that support somatic cells after GenX exposure, we identified the DEGs of each somatic cell type. Most DEGs were specific in different somatic cell types, of which 16 up-regulated DEGs were shared in more than two cell types, indicating the existence of commonality (Fig. 5A). These DEGs were enriched in stress responses such as “regulation of DNA-templated transcription in response to stress” and “negative regulation of response to endoplasmic reticulum stress”, inflammatory pathways such as “TNF signaling pathway” and apoptosis (Fig. 5B). The listed example genes *Fos* and *Gadd45b* indicate that GenX exposure might induce up-regulation of somatic cell-related inflammatory signals leading to cell apoptosis (Fig. 5C). Among the down-regulated somatic DEGs, 37 DEGs were shared in more than two cell types (Fig. 5D). These down-regulated DEGs were enriched in some cell adhesion biological processes and pathways, such as “focal adhesion”, “ECM-receptor interaction” and “adherens junction” (Fig. 5E). The example genes *Atp5b* and *Gpx1* listed suggest that GenX exposure may attenuate the adhesion capacity of the associated somatic cells (Fig. 5F).

### GenX exposure increased abnormal macrophage differentiation

**Fig. 6:**
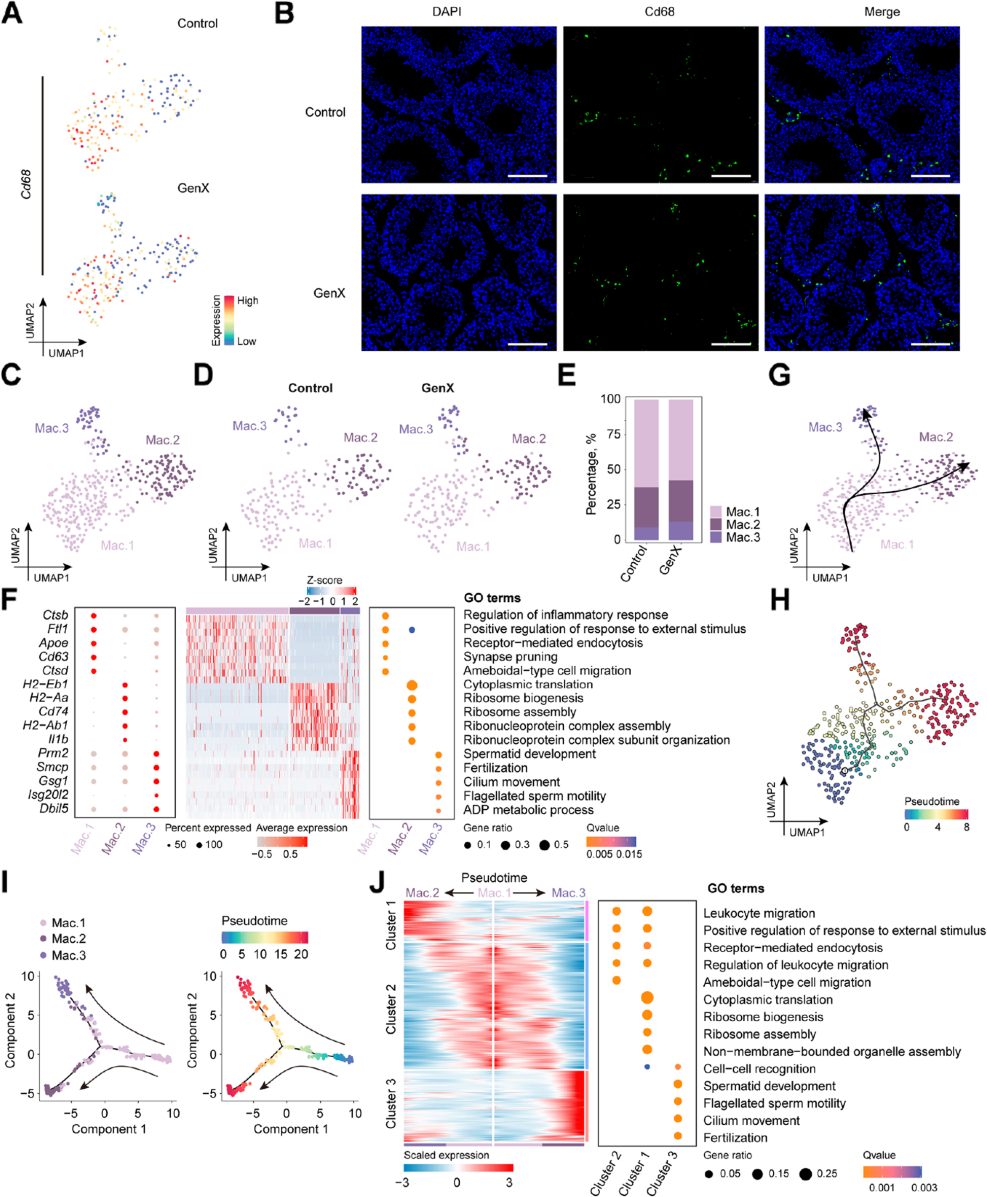
Cellular and functional changes in macrophages after GenX exposure. **A** UMAP visualization showing the expression of *Cd68* gene in macrophages of GenX-exposed and control groups, respectively. **B** Immunofluorescence staining of Cd68 protein in mouse testis tissue sections. Scale bar = 100 μm. **C** UMAP visualization showing the re-clustered macrophage subclusters in mouse testis. Cells from different mouse testes colored by macrophage subclusters. **D** UMAP visualization showing different macrophage subclusters in the GenX-exposed and control groups, respectively. **E** Stacked bar plot showing the changes in cell proportions of macrophage subclusters in mouse testis after GenX exposure. **F** Dot plot and heatmap showing the top DEGs in different macrophage subclusters, as well as the GO biological process terms enriched by the corresponding macrophage subcluster DEGs. Each row of the heatmap represents a gene and each column represents a macrophage. **G** Macrophage differentiation trajectories in mouse testis predicted using Slingshot. The black curve representing the predicted pseudotime trajectory and the arrow representing the direction. **H** Predicted macrophage differentiation trajectories colored with pseudotime score using Monocle3. **I** Predicted macrophage differentiation trajectories colored by cell subclusters, and pseudotime score using Monocle2. Green representing an immature cell stage and red representing a mature cell stage. **J** Heatmap showing the gene expression profiles of 3 clusters along two diverse differentiation trajectories of macrophages based on the DEGs during differentiation, with blue indicating low levels and red indicating high levels of expression. Top GO biological process terms enriched by the corresponding cluster DEGs were shown on the right

Macrophages play an important role in regulating inflammatory responses [34]. To explore the changes in macrophages after GenX exposure, we focused on macrophages identified in mouse testis (Fig. 6A and B). Based on differences in gene expression, these macrophages were further subdivided into three subclusters (Fig. 6C), that is, these subclusters all expressed classical marker genes of macrophages, but at the same time expressed different gene sets (Additional file 3). Notably, the proportion of Mac. 3 subcluster was visibly increased after GenX exposure (Fig. 6D and E). GO enrichment analysis of DEGs in different macrophage subclusters showed that the Mac. 1 subcluster was enriched in biological processes such as “regulation of inflammatory response” and “receptor-mediated endocytosis” (Fig. 6F), and was a major functional subcluster of macrophages. Mac. 2 was enriched in “cytoplasmic translation” and “ribosome biogenesis”, while Mac. 3 were enriched for biological processes associated with “spermatid development” and “cilium movement” which were obviously abnormal. Pseudotime analysis was used to construct the developmental trajectory of macrophages, and it was found that both Mac. 2 and Mac. 3 were differentiated from Mac. 1 (Fig. 6G–I). The DEGs of Mac. 1 differentiation to Mac. 2 and Mac. 3 showed dynamic changes along the pseudotime and could be divided into three clusters (Fig. 6J). Among them, the DEGs in cluster 3 increased significantly along the trajectory from Mac. 1 to Mac. 3, and were also enriched in related biological processes such as “spermatid development” and “cilium movement”, suggesting abnormal differentiation of macrophages. Overall, GenX exposure increased abnormal macrophage differentiation, which may be responsible for the disturbed testicular inflammatory response.

### GenX exposure attenuated fibroblast adhesion

**Fig. 7:**
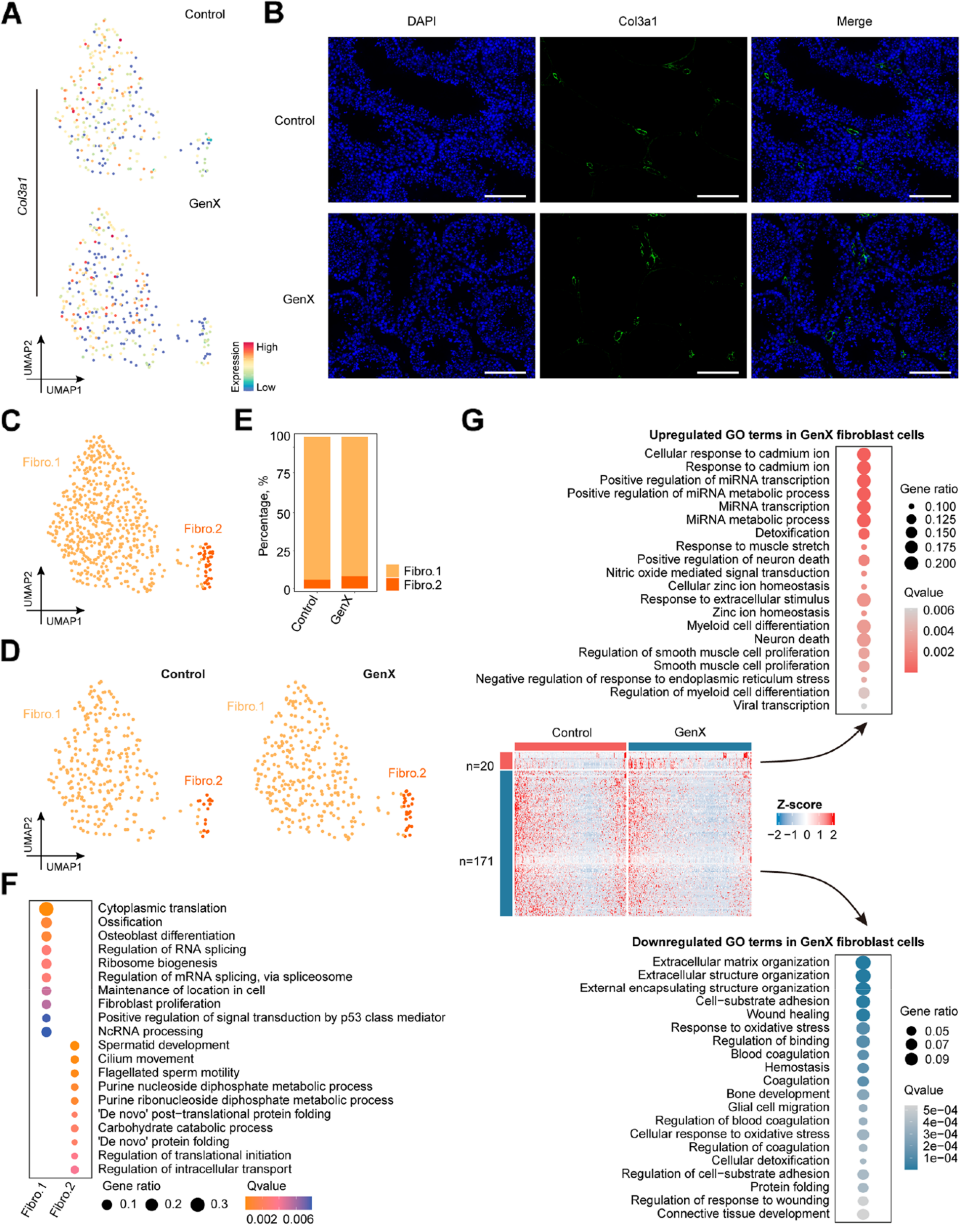
Cellular and functional changes in fibroblasts after GenX exposure. **A** UMAP visualization showing the expression of *Col3a1* gene in fibroblasts of GenX-exposed and control groups, respectively. **B** Immunofluorescence staining of Col3a1 protein in mouse testis tissue sections. Scale bar = 100 μm. **C** UMAP visualization showing the re-clustered fibroblast subclusters in mouse testis. Cells from different mouse testes colored by fibroblast subclusters. **D** UMAP visualization showing different fibroblast subclusters in the GenX-exposed and control groups, respectively. **E** Stacked bar plot showing the changes in cell proportions of fibroblast subclusters in mouse testis after GenX exposure. **F** Top GO biological process terms enriched by DEGs of different fibroblast subclusters. **G** Heatmap showing up-regulated and down-regulated DEGs in fibroblasts after GenX exposure. Each row represents a gene and each column represents a fibroblast. Dot plots listing the top 20 up- or down-regulated GO terms enriched in the DEGs

The integrity of the testicular structure is essential for maintaining spermatogenesis [35]. After GenX exposure, the loose morphology of testicular tissue was consistent with the weakened cell adhesion ability of the above-mentioned shared down-regulated DEGs in support somatic cells (Fig. 1D). Among these somatic cells, fibroblasts were selected for a focused analysis due to their most significantly downregulated DEGs (Fig. 7A and B). Based on the differences in gene expression, these fibroblasts could be further subdivided into 2 subclusters (Fig. 7C, Additional file 4). Notably, the proportion of Fibro. 2 subcluster slightly increased after GenX exposure (Fig. 7D and E). GO enrichment analysis of DEGs in the two fibroblast subclusters showed that the Fibro. 1 subcluster was enriched in biological processes such as “maintenance of location in cell” and “fibroblast proliferation” (Fig. 7F), and was the main functional subcluster of fibroblasts. Interestingly, the DEGs of Fibro. 2 were similar to those of the above-mentioned Mac. 3 subcluster (Fig. 6F), and were also related to “spermatid development” and “cilium movement”, representing an obviously abnormal process. Comprehensive analysis of differential gene expression revealed 20 up-regulated DEGs and 171 down-regulated DEGs in fibroblasts after GenX exposure (Fig. 7G). The up-regulated DEGs were enriched in biological processes related to stimulus response, such as “cellular response to cadmium ion”, “response to extracellular stimulus” and “negative regulation of response to endoplasmic reticulum stress”. While the down-regulated DEGs were enriched for cell adhesion, such as “extracellular matrix organization”, “cell-substrate adhesion” and “regulation of binding”. Collectively, the data suggest that GenX exposure leads to decreased fibroblast adhesion and may ultimately cause testicular structural damage.

### GenX disorganized the somatic-germline interactions in mouse testis

**Fig. 8:**
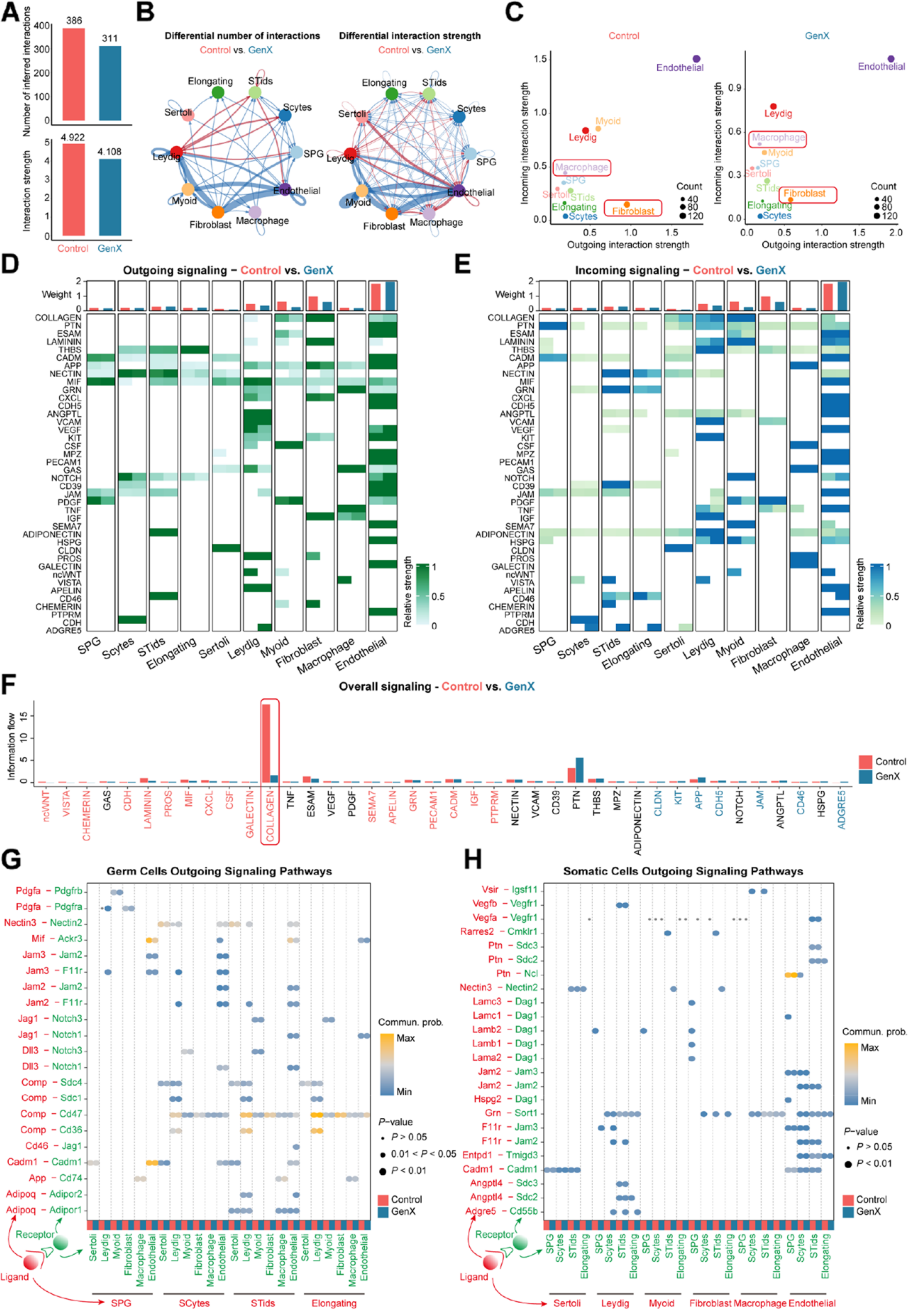
Interactions between testicular cells in mice after GenX exposure. **A** Bar plot showing the number and strength of cell–cell interactions in mouse testes after GenX exposure compared to control. The Control label refers to the interaction between testicular cells of the control mice, and the GenX label refers to the interaction between testicular cells of the mice after GenX exposure. **B** Network plot for the differential number of interactions or interaction strengths in the communication network between different mouse testicular cell types after GenX exposure compared to control. **C** Outgoing and incoming communication probabilities and number of interactions in different cell types of mouse testis. **D** and **E** Heatmap showing the relative strength of outgoing (**D**) and incoming (**E**) signals in each testicular cell type after GenX exposure compared with the control group. The bar plot above showing the relative strength of overall signals in each testicular cell type. **F** Bar plot showing the overall strength of each signaling pathway after GenX exposure compared with the control group. **G** Dot plot showing outgoing signals from germ cells emitted to somatic cells. Dot color reflects communication probabilities and dot size represents computed *P*-values. Empty space means the communication probability is zero. *P*-values are computed from one-sided permutation test. **H** Dot plot showing outgoing signals from somatic cells emitted to germ cells

To investigate the interactions between somatic cells and germ cells in mouse testis after GenX exposure, we applied CellChat to identify pathway communication between different testicular cell types [27]. Then, we found that after GenX exposure, both the number and strength of interactions between different types of somatic cells and germ cells in mouse testis were significantly reduced (Fig. 8A and B). When comparing the outgoing and incoming signaling of all testicular cell types, we found that GenX exposure led to an increase in the outgoing and incoming signaling of macrophages (Fig. 8C), consistent with the expanded inflammatory response revealed by above analysis. Meanwhile, the outgoing and incoming signaling of fibroblasts were significantly reduced, and the level of COLLAGEN signaling pathway was drastically weakened (Fig. 8D–F), which was consistent with the decreased cell adhesion caused by GenX exposure revealed by above analysis. Next, we examined specific ligand and receptor signaling between somatic and germ cells (Fig. 8G and H). We found that GenX exposure increased multiple abnormal signaling from germ cells to Leydig cells, such as Pdgfa-Pdgfra and Jam3-F11r in SPG, Nectin3-Nectin2 and Jam3/2-F11r in Scytes. Furthermore, multiple signaling interactions between somatic cells and germ cells were lost. For example, Lama2-Dag1, Lamb1/2-Dag1, and Lamc3-Dag1 from fibroblasts to SPG, and Grn-Sort1 from fibroblasts to Scytes, STids, and Elongating. Altogether, these results suggest that GenX disrupts somatic-germline interactions in mouse testis.

## Discussion

The reproductive toxicity of PFOA has been widely reported in previous studies [36, 37], but limited studies have evaluated the reproductive toxicity of its alternative GenX. This study confirmed that GenX exposure damages the testicles, which is consistent with previous studies showing that GenX exposure can disrupt the blood-testis barrier and lead to male reproductive toxicity [14]. To understand the potential mechanisms of male reproductive toxicity after GenX exposure, we performed scRNA-seq analysis of testicular tissue exposed to GenX and characterized cell type-specific molecular changes in mouse testis associated with GenX exposure, highlighting the heterogeneity of different cell types in response to the consequences of GenX exposure.

Our results showed that among all germ cells, GenX had the greatest adverse effect on Elongating. During normal spermatogenesis, accurate packaging of the paternal genome is critical for subsequent successful fertilization and embryo formation [38]. While elongating spermatids requires major chromatin remodeling to eliminate nucleosomal DNA supercoiling, which results in transient DNA strand breaks [39, 40]. Exposure to GenX may impair this process. However, since these sperm cells have limited active DNA repair capacity and only the error-prone non-homologous end joining repair pathway is available [41], GenX damage could eventually persist and cause male reproductive disorders.

Although the effect of GenX exposure on SPG was not as great as that on Elongating, we still found that it disrupted the balance between undifferentiated SSCs and differentiated SSCs. Undifferentiated SSCs with stem cell regeneration capacity are essential for the production of mature sperm and male fertility [42]. Functional analysis of up- and down-regulated DEGs in undifferentiated and differentiated SSCs indicated that, although the ratio of undifferentiated to differentiated SSCs was not altered in this study, GenX exposure may lead to the loss of the undifferentiated stem cell population, ultimately resulting in reduced spermatogenesis efficiency and subsequent stem cell exhaustion, as well as a gradual decline in male fertility [43, 44].

In addition to the germ cells, somatic cells are equally important in spermatogenesis due to their supporting roles. Interestingly, we found that inflammatory response DEGs shared among different testicular somatic cells were up-regulated after GenX exposure, which seems consistent with previous findings in aged testis [15, 16]. As a hallmark of aging, inflammation is well-known in many types of organs [45, 46]. While macrophages play an important role in regulating inflammatory responses [34]. The increased number of macrophages in aged mouse testes is consistent with the increased proportion of macrophages in the testes of mice exposed to GenX in this study, again implying that GenX exposure could lead to decreased fertility.

Moreover, oxidative stress and inflammation may impair spermatogenesis by downregulating tight junction occludin through activation of the p38 MAPK pathway, disrupting the integrity of the epithelial barrier [47, 48]. Heat shock proteins inhibit or neutralize oxidative stress-induced cell damage by synergizing with the antioxidant system [49]. As a result, oxidative stress activates nuclear factor kappa B (NF-kB) through p38 MAPK and others, thereby upregulating the TNF signaling pathway [50]. PFOA has been previously shown to cause oxidative damage to target organs by activating peroxisome proliferator-activated receptors α and γ (PPAR α and γ) [51, 52]. GenX can also activate human and rat PPARα and PPARγ variants in vitro and is one of the most potent PPARα-inducing PFAS tested to date [20], which may be a possible reason for the adverse effects of GenX.

Among the down-regulated somatic DEGs, the attenuation of cell adhesion function attracted our attention. Cell adhesion is crucial for spermatogenesis and is involved in regulating not only the cell–cell physical interactions but also other cellular events such as cell signaling and cell polarity [53]. In particular, different cell types in the testis need to be anchored in specific locations to create niches with specific functions. Specifically, activation of Jam3-F11r plays a role in multiple processes related to cell motility, including tight junction signaling and regulation of epithelial-mesenchymal transition by growth factors, which could modulate integrin signaling and promote cell motility [54, 55]. Studies have shown that weakened cell adhesion can lead to testicular structural remodeling, thereby destroying the blood-testis barrier and causing damage to germ cells [56, 57]. As for perivascular fibroblasts, maintaining their homeostasis can regulate blood microcirculation in the testis, promote angiogenesis, and participate in immune response [58, 59].

In this study, we observed adverse effects of GenX exposure on mouse testis, including spermatogenesis and somatic cell dynamics. Notably, the exposure level of mice in this study was higher than levels found in food and human exposure in the environment. Previous studies have shown that the concentration of GenX in urine samples from the general population is 0.07–0.4 μg/L [60], while the concentration of GenX in serum samples of highly exposed populations is 0.13–1.72 μg/L [61]. In this study, the concentrations of GenX in mouse serum in the 12.5 mg/kg/d and 62.5 mg/kg/d dose groups were 16.53 μg/mL and 34.48 μg/mL, respectively. Calculated a per unit body weight basis, the equivalent dose for mice is 12.3 times that for humans [21], and the GenX concentration in the 12.5 mg/kg/d dose group of mice was 781.3 times higher than that in human samples. However, considering that humans are exposed to GenX for a long time in the environment, it is unreasonable to compare exposure doses without considering the exposure time, so more reproductive toxicology studies on long-term chronic exposure to GenX are needed. Meanwhile, this study’s single-cell analysis involved only 2 biological replicates in each group, and the number of samples should be expanded further in future studies to obtain more comprehensive results. In addition, recent studies have demonstrated that adverse effects on paternal sperm can affect offspring through small RNA and epigenetic regulation [62, 63]. The transgenerational effects of additional exposure to GenX require further investigation to fully evaluate the male reproductive risks of GenX. In the livestock sector, such as the pig industry, considering its adverse consequences on male reproduction, it is urgent to pay attention to the contamination of GenX in pig farms, especially boar farms, and strengthen the monitoring of feed and environment.

## Conclusion

In conclusion, this study revealed complex cellular dynamics and transcriptome changes in mouse testis after GenX exposure, providing valuable insights into the potential mechanisms behind its male reproductive toxicity. Our findings highlight the need for increased scrutiny of GenX and GenX-like PFOA alternative compounds to safeguard reproductive health and inform regulatory policy.

## Supplementary Information

Additional file 1: Table S1. Differentially expressed genes in 10 major cell types.

Additional file 2: Table S2. Differentially expressed genes in 4 germ cell types.

Additional file 3: Table S3. Differentially expressed genes in 3 macrophage subclusters.

Additional file 4: Table S4. Differentially expressed genes in 2 fibroblast subclusters.

## Data Availability

The raw sequence data reported in this paper have been deposited in the Genome Sequence Archive [64] in National Genomics Data Center [65], China National Center for Bioinformation / Beijing Institute of Genomics, Chinese Academy of Sciences (GSA: CRA017742) that are publicly accessible at https://ngdc.cncb.ac.cn/gsa.

## Abbreviations

DEGs: Differentially expressed genes
Elongating: Elongating spermatids
GO: Gene Ontology
H&E: Hematoxylin and eosin
HFPO-DA/GenX: Hexafluoropropylene oxide dimer acid
HVGs: Highly variable genes
KEGG: Kyoto Encyclopedia of Genes and Genomes
PAS: Periodic acid-Schiff
PFAS: Per- and polyfluoroalkyl substances
PFOA: Perfluorooctanoic acid
scRNA-seq: Single-cell RNA sequencing
Scytes: Spermatocytes
SEM: Standard error of the mean
SNN: Shared nearest neighbor
SPF: Specific pathogen-free
SPG: Spermatogonia
SSCs: Spermatogonial stem cells
STids: Round spermatids
UMAP: Uniform manifold approximation and projection
